# Seminal plasma untargeted metabolomic and lipidomic profiling for the identification of a novel panel of biomarkers and therapeutic targets related to male infertility

**DOI:** 10.3389/fphar.2023.1275832

**Published:** 2023-09-27

**Authors:** Serena Correnti, Mariaimmacolata Preianò, Annalisa Fregola, Fabia Gamboni, Daniel Stephenson, Rocco Savino, Angelo D’Alessandro, Rosa Terracciano

**Affiliations:** ^1^ Department of Health Sciences, Magna Græcia University, Catanzaro, Italy; ^2^ Urogyn Biotech S. R. L., Catanzaro, Italy; ^3^ Department of Biochemistry and Molecular Genetics, University of Colorado Anschutz Medical Campus, Aurora, CO, United States; ^4^ Department of Medical and Surgical Sciences, Magna Græcia University, Catanzaro, Italy; ^5^ Department of Experimental and Clinical Medicine, Magna Græcia University, Catanzaro, Italy

**Keywords:** male infertilty, seminal plasma, metabolomics, lipidomics, biomarkers, reproductive medicine, mass spectrometry

## Abstract

Male infertility occurs approximately in about 50% of all infertility cases and represents a serious concern worldwide. Traditional semen analysis alone is insufficient to diagnose male infertility. Over the past two decades, advances in omics technologies have led to the widespread application of metabolomics profiling as a valuable diagnostic tool for various diseases and disorders. Seminal plasma represents a rich and easily accessible source of metabolites surrounding spermatozoa, a milieu that provides several indispensable nutrients to sustain sperm motility and fertilization. Changes of metabolic profiles in seminal plasma reflect male reproductive tract disorders. Here, we performed seminal plasma metabolomics and lipidomics profiling to identify a new pattern of biomarkers of male infertility. Seminal plasma samples from unfertile subjects (*n* = 31) and fertile controls (*n* = 19) were analyzed using an untargeted metabolomics/lipidomics integrated approach, based on Ultra-High-Pressure Liquid Chromatography–tandem Mass Spectrometry. Partial Least Squares-Discriminant Analysis showed a distinct separation between healthy fertile men and infertile subjects. Among the 15 selected candidate biomarkers based on Variable Importance in Projection scores, phosphatidylethanolamine (PE) (18:1; 18:1) resulted with the highest score. In total, 40 molecular species showed statistically significant variations between fertile and infertile men. Heat-map and volcano plot analysis indicated that acylcarnitines, phosphatidylserine (PS) (40:2) and lactate were decreased, while PE (18:1; 18:1), Phosphatidic acid (PA) (O-19:2; 18:1), Lysophosphatidylethanolamine (LPE) (O-16:1) and Phosphatidylcholine (PC) (O-16:2; 18:1)-CH3 were increased in the infertile group. The present study is the first one to analyze the metabolomics/lipidomics dysregulation in seminal plasma between fertile and infertile individuals regardless of sub-infertility condition. Association of several metabolites/lipids dysregulation with male infertility reinforced data of previous studies performed with different approaches. In particular, we confirmed significantly decreased levels of PS and carnitines in infertile patients as well as the positive correlation with sperm motility and morphology. If validated on a larger prospective cohort, the metabolite biomarkers of infertility in seminal plasma we identified in the present study might inform novel strategies for diagnosis and interventions to overcome male infertility.

## 1 Introduction

Infertility is a worldwide health problem characterized by the inability to achieve conception despite regular unprotected sexual intercourse for a duration of 12 months or more (https://www.who.int/news-room/fact-sheets/detail/infertility, accessed on 9 9 August 2023). Around 15% of couples of reproductive age are affected, with male factors contributing to around 50% of cases independently or in conjunction with female factors (Agarwal et al., 2021). Currently, the clinical diagnosis and prediction of male infertility primarily rely on semen analyses performed in accordance with the guidelines established by the World Health Organization, 2020; Practice Committee of the American Society for Reproductive Medicine, 2015). These analyses involve the assessment of various sperm parameters, including sperm count, motility and morphology. By evaluating these parameters, male infertility forms are commonly classified into qualitative abnormalities such as asthenozoospermia, teratozoospermia and necrospermia, as well as quantitative abnormalities like azoospermia, cryptozoospermia and oligozoospermia (Boitrelle et al., 2021). Although semen analysis provides useful information, alone it does not provide a comprehensive evaluation of male fertility potential or an accurate differentiation between fertile and infertile individuals (Agarwal et al., 2021). In fact, semen analysis is not able to effectively identify underlying contributing factors or elucidate the molecular and pathophysiological mechanisms of male reproductive disorders (Preianò et al., 2023). In particular, a more in-depth analysis is required for the idiopathic infertility (i.e., a condition whose cause remains unknown), which affects about 30% of men showing impaired sperm parameters without an identifiable cause of infertility, with normal findings on genetic and laboratory testing and physical examination (Panner Selvam et al., 2019; Agarwal et al., 2021). Additionally, a condition defined as unexplained male infertility accounts from 15% to 40% of men who are infertile despite showing normal medical history, normal sperm parameters and normal physical examination (Corsini et al., 2023). Therefore, both idiopathic and unexplained male infertility severely limit therapeutic treatment approaches to rescue fertility. Consequently, there is a critical need for non-invasive, rapid and more comprehensive diagnostic approaches to enhance the clinical management of male infertility (Mumcu et al., 2020).

Metabolomics and lipidomics have emerged as comprehensive approaches to identify specific biomarkers associated with reproductive disorders (Aiello et al., 2016; Aderemi et al., 2021; Panner Selvam et al., 2021).

Research approaches focusing on single omics levels have so far yielded limited results. While many contributing factors have been identified with these methods, the complex pathomechanisms of male infertility remain unclear. For this purpose, an integrated multi-omics approach may be more beneficial in unravelling the underlying disease mechanism, offering a more holistic analysis of the disease and providing enhanced screening methods and treatment options (Wagner et al., 2023).

In particular, metabolomics and lipidomics, by providing a direct signature of biochemical activity, are broadly acknowledged to be the omics which offer the closest representation of the phenotype, making them particularly informative in understanding cellular responses to external stimuli or pathological conditions (i.e., reproductive disorders) (Guijas et al., 2018; Blaurock et al., 2022).

In addition, seminal plasma (SP), the fluid portion of semen, has emerged as a promising, readily available and easily accessible matrix for metabolomic analysis in the assessment and diagnosis of male infertility (Blaurock et al., 2022; Preianò et al., 2023). SP is composed of secretions from testes, epididymis and male accessory glands (Owen and Katz, 2005). Due to its complex molecular composition including lipids, glycans, inorganic ions, metabolites, cell-free DNA, RNA, microRNAs, peptides, proteins, and oligosaccharides (Samanta et al., 2018), SP provides a metabolic support for spermatozoa protecting them from the moment of ejaculation until subsequent fertilization (Rodriguez-Martinez et al., 2009; Panner Selvam et al., 2019). Given these characteristics, SP is a suitable noninvasive sample for the direct understanding of the dysregulated metabolism in male reproductive disorders (Qiao et al., 2017). The identification and characterization of various biomarkers in SP hold significant implications not only for the diagnosis but also for potential treatment of male infertility (Drabovich et al., 2014).

Mass spectrometry (MS)-based metabolomics offers ultra-high sensitivity, a wide dynamic range and the ability to detect small variations in metabolic biomarkers associated with male infertility (Zhao et al., 2018; Li et al., 2020). However, the main findings originating from several of these investigations yet need to be validated (Blaurock et al., 2022) The highly dynamic nature of the metabolome and the variety of technical and biological factors require acquisition of more harmonized and reliable data (Blaurock et al., 2022) to unveil distinct metabolic patterns associated with male infertility.

In this study, we used an Ultra-high-pressure liquid chromatography (UHPLC)-MS based metabolomics approach to provide new insights into the metabolic and signaling pathways responsible for male infertility. In particular, we performed a multi-tiered analysis of the metabolomics and lipidomics data: first, we processed all combined data, regardless of the specific subfertility condition, pooling all the subjects with fragmented and dissimilar parameters of infertility as determined by semen analysis and comparing them against fertile controls. This analysis identified new sensitive biomarkers of male infertility diagnosis, that are not specific for a specific infertility sub-group. The ability of this platform for capturing a wider integrated panel of SP lipids and metabolites might offer new insights to address future prospective validation studies on larger male infertile cohorts, which may overcome the biased information obtained from classical semen analysis.

## 2 Materials and methods

### 2.1 Patient recruitment

Volunteer unfertile subjects (*n* = 31) and fertile controls (*n* = 19) were enrolled in accordance with the Declaration of Helsinki, upon approval by the Ethics Committee of MAGNA GRAECIA UNIVERSITY and MATER DOMINI HOSPITAL (protocol code 2014.39, date of approval 16 April 2014). Before participation, all individuals signed informed written consent and were provided with a questionnaire to obtain information on age, smoking habits, alcohol use (regular, irregular, or total abstinence), and use or abuse of other substances and drugs (yes or no). For this analysis, only the initial sperm evaluation was considered. Participants with a history of vasectomy, orchitis, testicular trauma, sexually transmitted disease, varicocele, inguinal hernia operation, or cryptorchism were excluded from the study. Participants were instructed to collect semen samples into sterile containers following a period of 3–5 days of sexual abstinence. All samples were processed and analyzed anonymously. The detailed clinical characteristics of the subjects enrolled in this study are summarized in Table 1.

**TABLE 1:** Clinical characteristics of the subjects enrolled in the study.

Patient features (mean ± SD)	NZ n = 19	AT n = 4	OAT n = 6	TZ n = 13	OZ n = 2	AZ n = 3	OA n = 3
Age	28 ± 7.4	40.5 ± 3.4	32.3 ± 5.8	34.1 ± 9	33 ± 14.1	34 ± 7.21	44 ± 11.3
Ejaculated volume (mL)	3.6 ± 1.7	3 ± 1	2.5 ± 0.8	3.8 ± 2.7	3.15 ± 0.2	2.5 ± 0.9	3.2 ± 1.3
pH	7.6 ± 0.4	7.8 ± 0.4	7.8 ± 0.4	7.5 ± 0.3	8.25 ± 0.2	7.5 ± 0.5	7.8 ± 0.3
Sperm count (milion)	214.9 ± 133.2	122.1 ± 64.3	3.1 ± 2.3	183.4 ± 54.4	20 ± 5.3	0	22.1 ± 14.3
Progressive motility (%)	48.9 ± 6.9	20 ± 7.8	12.2 ± 11.7	43.3 ± 5.4	40.5 ± 7.8	0	16.3 ± 11.7
Total motility (%)	61.4 ± 6.9	39.3 ± 9.9	17.8 ± 9.4	58.4 ± 6.3	53.5 ± 2.1	0	27.7 ± 19.6
Normal morphology (%)	7.2 ± 3.2	1.8 ± 1.3	0.7 ± 0.4	2.4 ± 1	4.25 ± 0.3	0	5.3 ± 0.6
Total protein conc. (mg/mL)	54.7 ± 11.3	46.9 ± 11.6	45.1 ± 7.6	53.8 ± 14	51.7 ± 29.7	52.1 ± 19.5	43 ± 6.2

NZ, Normozoospermic subject, a fertile man with all semen parameters within the acceptable reference values provided by the WHO 2010 guidelines (total sperm concentration ≥39 milion, sperm progressive motility ≥32% and normal sperm morphology ≥4%); AT, Asthenoteratozoospermic patient, an infertile man with alteration of sperm progressive motility (<32%) and normal morphology (<4%); OAT, Oligoasthenoteratozoospermic patient, an infertile man with alteration of sperm number (<39 milion), progressive motility (<32%) and normal morphology (<4%); TZ, Teratozoospermic patient, an infertile man with alteration of normal morphology (<4%); OZ, Oligozoospermic patient, an infertile man with alteration of sperm number (<39 milion); AZ, Azoospermic patient, an infertile man without spermatozoa in the ejaculate; OA, Oligoasthenozoospermic patient, an infertile man with alteration of sperm number (<39 milion) and sperm progressive motility (<32%).

### 2.2 Preparation of SP

SP samples were processed as previously described (Correnti et al., 2022). Briefly, each ejaculate was allowed to liquefy for 15–30 min at 37°C. Semen parameters were assessed referring to the World Health Organization guidelines 2010 (Cooper et al., 2010). After the complete liquefaction of the coagulum, liquefied sample from each subject was divided into two aliquots. In one aliquot a protease inhibitor cocktail (PIC) was immediately added in a 1:100 v/v ratio. We apply a rigorous analytical protocol considering that given their degradative role, proteases indirectly might influence protein activity and consequently the production of bioactive molecules as well as amino-acids analyzed in metabolomics analysis. Then, both aliquots were processed to obtain SP. In particular, each clinical sample was centrifuged at 150,00× g for 15 min at 4°C. The supernatant (representing the SP) resulted as a clear and fluid phase that was separated from pellets (composed by sperm cells), aliquoted, and stored at −80°C until use. Only aliquots with PIC were used for the analysis. Aliquots with and without PIC were also stored for future analysis aimed at investigating whether prolonged sample storage generates altered metabolites profiles. Protein concentration of SP was determined by the bicinchoninic acid (BCA) assay according to the manufacturer’s instructions.

### 2.3 Metabolites and lipid extraction

Metabolomics and lipidomics analysis were performed at the University of Colorado Anschutz Medical Campus Metabolomics Core. SP samples were thawed and metabolites and lipids extracted by adding chilled 5:3:2 methanol:acetonitrile:water (v/v/v) at 1:25 ratio, vortexed for 30 min at 4°C and centrifugated for 10 min at 18,000 rcf at 4°C. The supernatant was then separated from pellet and used for metabolomics and lipidomics analysis according to a previous study by Nemkov et al. (2019); Reisz et al. (2019).

### 2.4 UHPLC-MS metabolomics

Metabolomics analyses were performed using a Vanquish UHPLC combined with a high-resolution Q Exactive mass spectrometer (Thermo Fisher, Bremen, Germany) in negative and positive polarity modes according to a previous study of Nemkov et al. (2019). For each method, the LC utilized a Phenomenex C18 column at a flow rate of 0.45 mL/min with a 5-min gradient. Samples were resolved over a Kinetex C18 column (2.1 mm × 150 mm 1.7 µm; Phenomenex, Torrance, CA, United States) equipped with a guard column (SecurityGuardTM Ultracartridge—UHPLC C18 for 2.1 mm ID Columns, AJO-8782, Phenomenex, Torrance, CA, United States). A volume of 5 µL of sample was injected into the UHPLC-MS. Each sample was injected and run with two different chromatographic and MS conditions as follows: 1) using an aqueous phase (A) of water and 0.1% formic acid and a mobile phase (B) of acetonitrile and 0.1% formic acid when the MS was operated in positive ion polarity mode and 2) an aqueous phase (A) of water:acetonitrile (95:5) with 1 mM ammonium acetate and a mobile phase (B) of acetonitrile:water (95:5) with 1 mM ammonium acetate) when the MS was operated in negative ion mode. Samples were eluted from the column using either an isocratic elution of 5% B flowed at 250 μL/min and 25°C or a gradient from 5% to 95% B over 1 min, followed by an isocratic hold at 95% B for 2 min, flowed at 400 μL/min and 45°C. The UHPLC system was coupled online with a Q Exactive mass spectrometer scanning in Full MS mode at 70,000 resolution in the 60–900 *m/z* range, with 4 kV spray voltage, 15 sheath gas, and 5 auxiliary gas, operated in negative or positive ion mode (separated runs). Calibration was performed prior to analysis using the Pierce™ Positive and Negative Ion Calibration Solutions (Thermo Fisher Scientific). Samples were analyzed in randomized order and technical mixture as quality controls (generated by mixing 10 uL of all samples tested in this study) were injected every 10 runs and were used to qualify instrument performance. These samples were injected every 10 runs to ensure technical reproducibility, as gleaned by determining coefficients of variation (CV, calculated by dividing standard deviations for each metabolite by the mean across all tech mixes) for each reported metabolite <20% for chromatographic retention times and peak areas. Metabolomics data were collected using Thermo Fisher Scientific Xcalibur software (v.4.1.31.9). LC-MS raw files were converted to mzXML file format using MSconvert v.3.0.20315–7da487568 (ProteoWizard). Data analysis and peak picking (including ^13^C-labeled isotopologues of internal standards) were performed via Maven (1.4.20-dev-772). The MAVEN software platform provides tools for peak picking, feature detection, metabolite assignment using the Kyoto Encyclopedia of Genes and Genomes (KEGG) pathway database. A coefficient of variation <30% in the technical mixtures was used as a threshold for reporting metabolites. Data normalization to protein concentration was performed.

### 2.5 UHPLC-MS lipidomics

Lipidomics analyses were performed using a Vanquish UHPLC system coupled online to a high-resolution Q Exactive mass spectrometer (Thermo Fisher, Bremen, Germany) (Reisz et al., 2019). Samples were resolved over an ACQUITY HSS T3 column (2.1 × 150 mm, 1.8 µm particle size (Waters, MA, United States) using an aqueous phase (A) of 25% acetonitrile and 5 mM ammonium acetate and a mobile phase (B) of 90% isopropanol, 10% acetonitrile, and 5 mM ammonium acetate. Samples were eluted from the column using either the solvent gradient: 0–9 min 30%–100% B at 0.325 mL/min; hold at 100% B for 3 min at 0.3 mL/min, and then decrease to 30% over 0.5 min at 0.4 mL/min, followed by a re-equilibration hold at 30% B for 2.5 min at 0.4 mL/min. The Q Exactive mass spectrometer (Thermo Fisher Scientific, San Jose, CA, United States) was operated independently in positive or negative ion mode, scanning in Full MS mode (2 μscans) from 150 to 1,500 m/z at 70,000 resolution, with 4 kV spray voltage, 45 sheath gas, and 15 auxiliary gas. For discovery mode untargeted lipidomics, dd-MS2 was performed at 17,500 resolution, AGC target = 1 × 105, maximum IT = 50 ms, and stepped NCE of 25, 35 for positive mode, and 20, 24, and 28 for negative mode. Calibration was performed prior to analysis using the Pierce™ Positive and Negative Ion Calibration Solutions (Thermo Fisher Scientific). Samples were analyzed in randomized order and technical mixture or quality controls (generated by mixing 10 uL of all samples tested in this study) were injected every 10 runs and were used to qualify instrument performance (CV<20%).

Discovery mode analysis was performed with standard workflows using Compound Discoverer 2.1 SP1 (Thermo Fisher Scientific, San Jose, CA, United States). From these analyses, metabolite IDs or unique chemical formulae were determined from high-resolution accurate intact mass, isotopic patterns, identification of eventual adducts (e.g., Na^+^ or K^+^, etc.) and MS2 fragmentation spectra against the KEGG pathway, HMDB, ChEBI, and ChEMBL databases. Additional untargeted lipidomics analyses were performed with the software LipidSearch 4.0 (Thermo Fisher, Bremen, Germany), which provides lipid identification on the basis of accurate intact mass, isotopic pattern, and fragmentation pattern to determine lipid class and acyl chain composition. Data normalization to protein concentration was then performed.

### 2.6 Statistical analysis

Statistical analyses were performed using a two-sided Student’s t-test on peak intensity with the level of statistical significance set to *p*-values of <0.05. MetaboAnalyst (5.0) was used to perform multivariate analyses including Partial Least Square-Discriminant Analysis (PLS-DA), Orthogonal partial least squares discriminant analysis (OPLS-DA) and hierarchical clustering analyses with heatmap representation of the top 50 metabolites. PLS-DA and OPLS-DA were conducted multivariate supervised methods to reduce the dimensionality of data and to detect the variables which show the ability to differentiate between groups. Box plot and correlation analysis (Spearman) were carried out in RStudio (Boston, Massachusetts, United States). The correlation analysis was performed in order to provide useful insights into the clinical importance and the potential diagnostic significance of the identified metabolites and lipids. ROC curve and cumulative ROC curve analysis were performed by MetaboAnalyst (5.0).

## 3 Results

### 3.1 Patients’ characteristics

**FIGURE 1:**
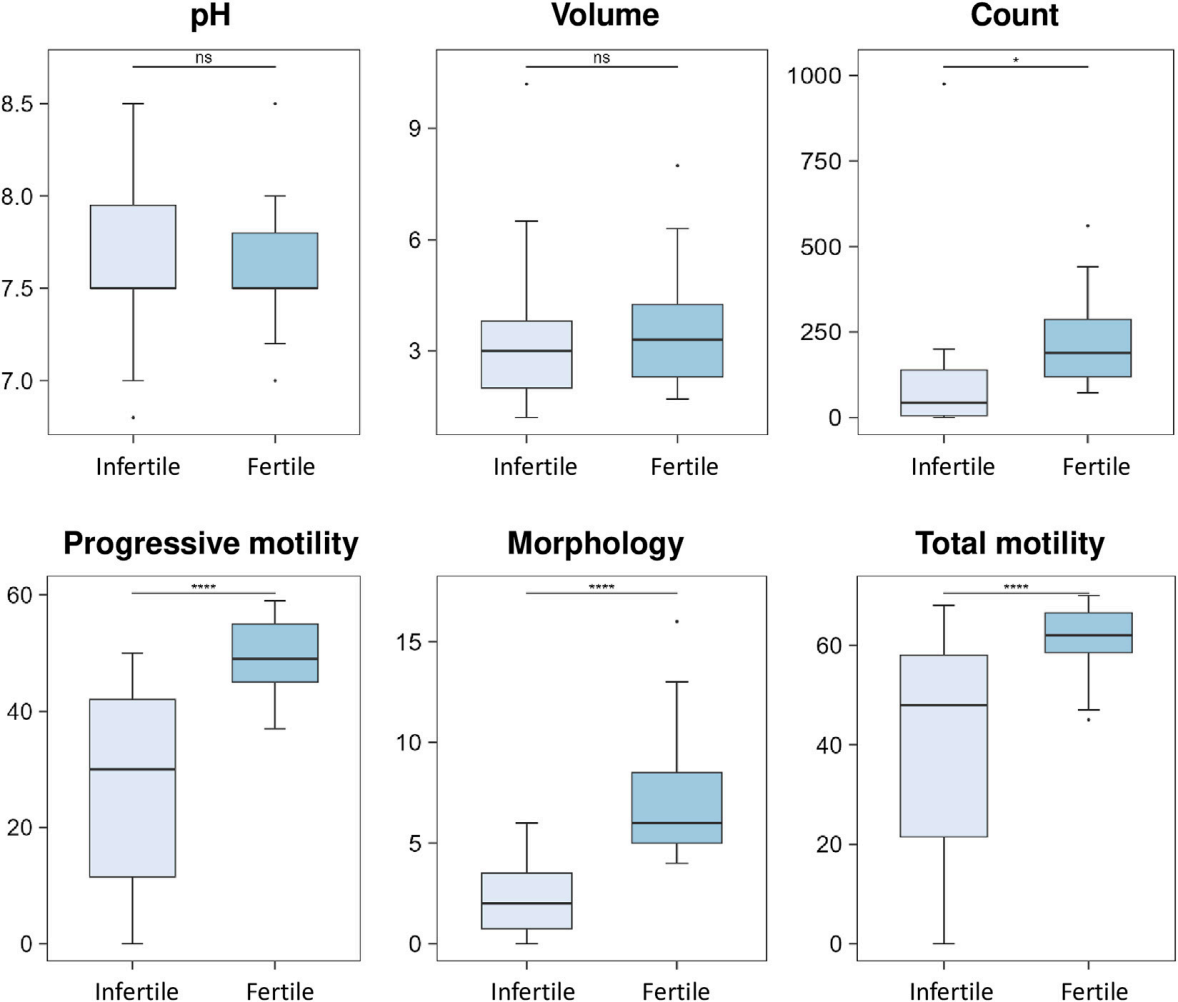
Box plot analysis of spermiogram parameters between fertile and infertile group. The box plots show the comparison of sperm parameters including pH, volume, count, progressive motility, morphology and total motility between fertile men and infertile patients. The *p-*values were calculated with Student t-test and the asterisks show the level of significance between the two groups; * *p*-values <0.05, **** *p*-values <0.0001, ns, not significant. Box plot analysis was performed by RStudio.

Table 1 shows the general clinical characteristics of the two groups of participants enrolled in this study including all semen parameters for semen subtype classification according to the World Health Organization (WHO) 2010 guidelines. The presence of statistically significant differences in the spermiogram parameters between the fertile and infertile groups was evaluated and the results are shown in the box plots (Figure 1). No statistically significant difference was found for pH and volume between fertile and infertile group (Figure 1). In contrast, statistically significant decreases were observed between groups with respect to sperm parameters, such as count (*p*-value < 0.05), progressive motility, total motility and morphology (*p*-value < 0.0001 - Figure 1), as expected per study design.

### 3.2 Metabolomics and lipidomics profiling of SP between fertile and infertile patients

To delve into the molecular underpinning of male infertility, we then performed an untargeted integrated metabolomics and lipidomics characterization of SP samples from fertile and infertile men.

Multivariate analyses of omics data were performed via PLS-DA and OPLS-DA, which were plotted to visually discriminate fertile and infertile groups and to identify the variables that contribute to explaining the variance across samples from each group (Rousseau et al., 2008). In particular, PLS-DA was employed to maximize class segregation and simplify interpretations identifying potential metabolic biomarkers. OPLS-DA is orthogonal to PLS-DA, in that it is a supervised pattern-recognition procedure that combines the theory of PLS-DA and orthogonal signal correction (OSC) to remove any unrelated variability to groups segregation; this algorithm discloses more subtle changes in the expression levels of specific metabolites by focusing on compounds accountable for the discrimination between classes (Trygg and Wold, 2002).

**FIGURE 2:**
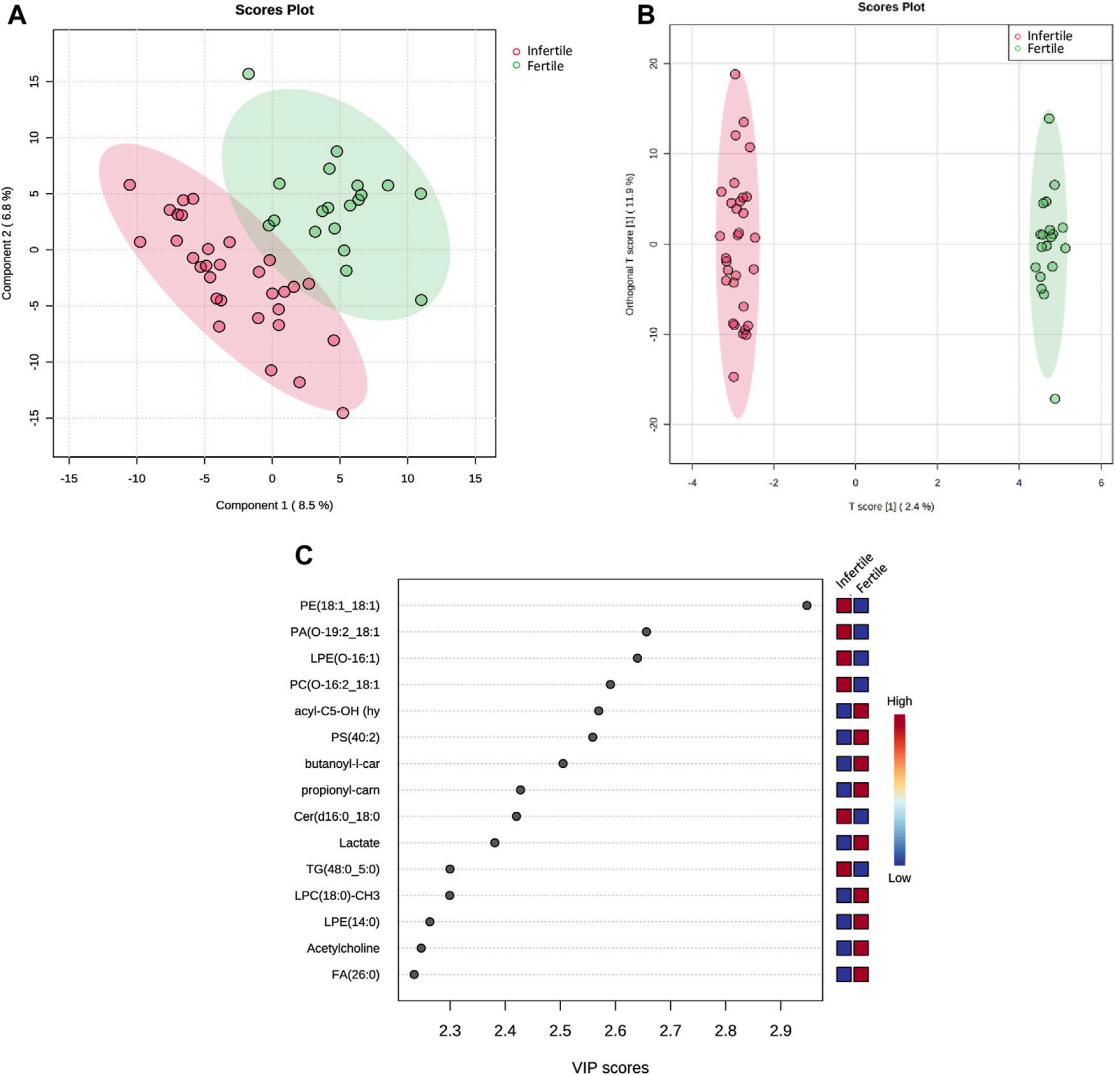
Multivariate statistical analysis of SP metabolic and lipidomic profiling between fertile and infertile men. **(A)** Partial least squares-discriminant analysis score plot (PLS-DA) and **(B)** Orthogonal partial least squares discriminant analysis (OPLS-DA) score plot show visual discrimination of the metabolome and lipidome profiles between infertile patients and fertile men. **(C)** VIP score plot, generated by PLS-DA, shows the top 15 metabolites and lipid compounds that differed in infertile patients vs*.* fertile men,. Colored boxes on right indicate the relative concentration of the corresponding compound; in particular blue indicates low concentration, while red represents high concentration. PLS-DA, OPLS-DA and VIP score plots were acquired by Metaboanalyst software.

Through PLS-DA model, a clear differentiation of infertile patients from fertile ones was achieved (Figure 2A). Then, the OPLS-DA model improved the clarity of class discrimination (Figure 2B), with principal component 1 in both analyses explaining ∼10% of the total variance. Two distinct separate clusters were visualized indicating that OPLS-DA satisfactorily segregates the metabolome and lipidome profiles of infertile patients from fertile normozoospermic controls. Infertile individuals (red dots) appear tightly clustered and cleanly separated in the plot from the fertile individuals (green dots).

Based on successful discrimination of the fertile and infertile groups, the search for the specific metabolites or lipid compounds that contributed to the differences between the two groups was performed.

In particular, the Variable Importance in the Projection (VIP) plot, generated from PLS-DA, was employed to assess the impact of each metabolite’s expression pattern on the classification and differentiation among samples (Figure 2C). A higher VIP score assigned to a metabolite indicates a more significant contribution of that particular metabolite in distinguishing between fertile men and infertile patients. PLS-DA VIP plot (Figure 2C) plotted for the top 15 metabolites with a VIP value >1 suggested that the separation between the infertile and fertile groups was based on the following compounds: Fatty acid (FA) (26:0), Acetylcholine, Lysophosphatidylethanolamine (LPE) (14:0), Lysophosphatidylcholine (LPC) (18:0)-CH3, Triglyceride (TG) (48:0; 5:0), Lactate, Ceramide (Cer) (d16:0; 18:0), butanoyl-l-carnitine (acyl-C4), propionyl-l-carnitine (acyl-C3), Phosphatidylserine (PS) (40:2), hydroxyisovalerylcarnitine (acyl-C5-OH), Phosphatidylcholine (PC) (O-16:2; 18:1)-CH3, LPE(O-16:1), Phosphatidic acid (PA) (O-19:2; 18:1), Phosphatidylethanolamine (PE) (18:1; 18:1).

**FIGURE 3:**
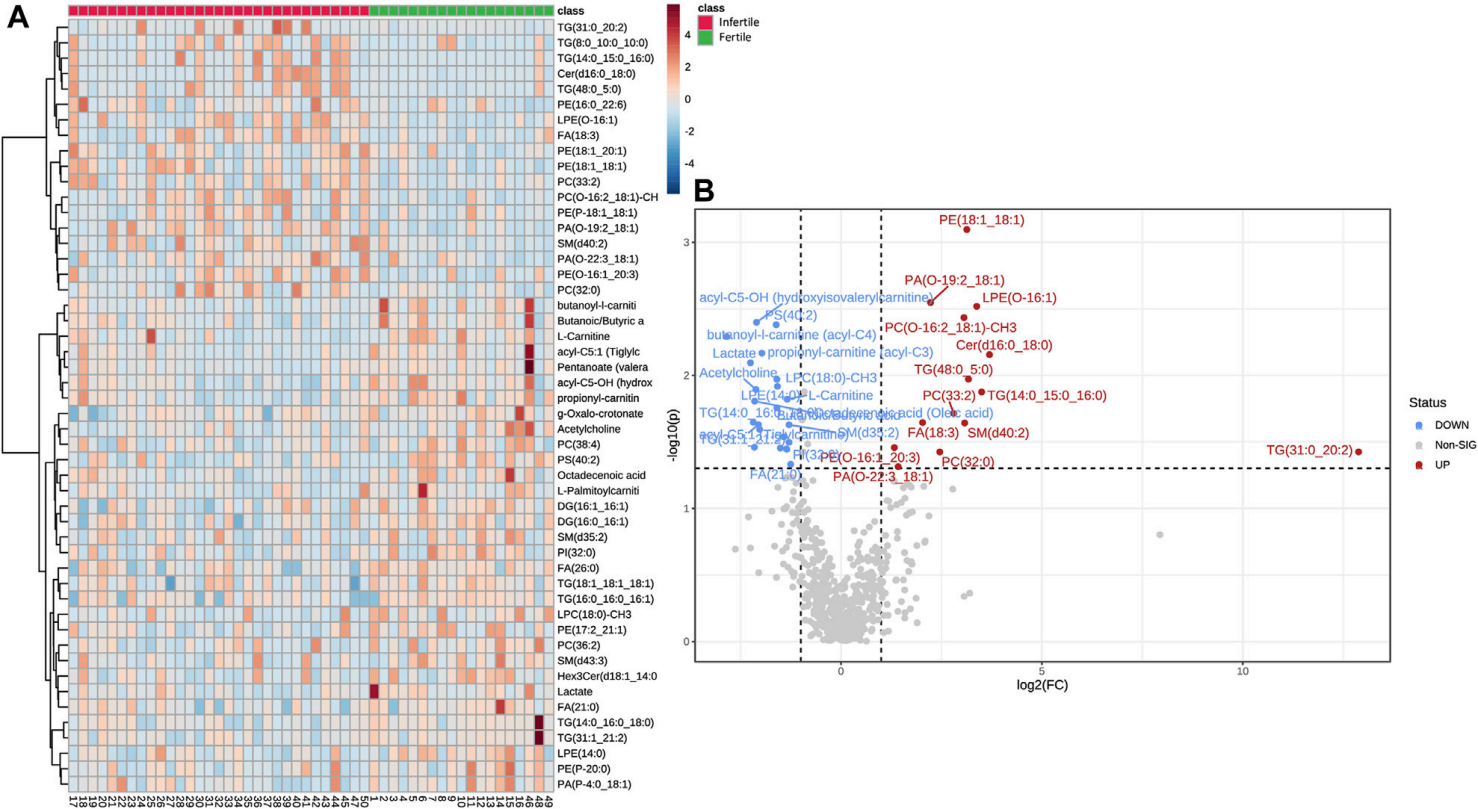
Differential abundances of metabolites and lipids in SP samples. **(A)** The Heat-map displays the level of relative expression of the top 50 metabolites and lipids in fertile men vs*.* infertile patients. Red indicates upregulation and blue indicates downregulation. The columns and rows represent experimental SP samples and metabolites, respectively. **(B)** Volcano plot shows metabolites and lipids compounds that differ significantly between the two groups, based on Fold change (Infertile/Fertile) and *p*-value. In particular, blue (Fold Change <0.67; *p*-value <0.05) or red dots (Fold Change >1.5; *p*-value <0.05) indicate the presence of significantly downregulated or upregulated metabolites, respectively. Gray dots were non-significantly different compounds. Heat-map and Volcano plot were acquired by Metaboanalyst software.

Hierarchical clustering was performed with results illustrated as a Heatmap (Figure 3A), showing the top 50 metabolites and lipids with their detailed relative expression levels and distinct segregation between fertile normozoospermic and unfertile groups.

The metabolomic and lipidomic data were further elaborated in the volcano plot in Figure 3B, which shows metabolites and lipid compounds that differ significantly between the two groups, based on a fold change greater than 1.5 or less than 0.67 (Infertile/fertile) and FDR-corrected *p*-values less than 0.05 (to account for multiple hypothesis testing).

Based on the results of the multivariate statistical analysis, a *t*-test (*p*-value <0.05) was used to check whether the relevant peaks from the PLS-DA VIP plot (VIP score >1) were statistically significant (Table 2). Statistically significant variations between fertile and infertile men were detected for 40 compounds (metabolites and lipids) as listed in Table 2.

**TABLE 2:** List of differential SP metabolites and lipids in infertile patients compared with fertile men.

Metabolite	*m/z*	Rt (s)	VIP-score	Fold change^a^	*p*-value	Metabolic pathway (biological function)	Class
PE(18:1; 18:1)	742.5392	3.41	2.9454	8.7642	0.0008	Glycerophospholipids metabolism	GPE
PA(O-19:2; 18:1)	716.559	3.12	2.6545	4.6846	0.0028	Phospholipids metabolism	PA
LPE(O-16:1)	436.2833	2.47	2.6381	10.397	0.0030	Glycerophospholipids metabolism	GPE
PC(O-16:2; 18:1)-CH3	726.5443	3.46	2.5892	8.3386	0.0037	Glycerophospholipids metabolism	GPC
Acyl-C5-OH	262.1650	0.73	2.568	0.23293	0.0040	Fatty acids metabolism	Fatty acylcarnitine
PS(40:2)	844.6062	2.95	2.5571	0.32708	0.0042	Phospholipids metabolism	GPS
Acyl-C4	232.1543	1.74	2.5033	0.13933	0.0051	Fatty acids metabolism (energy source)	Fatty acylcarnitine
Acyl-C3	218.1387	0.72	2.4262	0.25563	0.0068	Fatty acids metabolism (energy source and membrane stabilizer)	Fatty acylcarnitine
Cer(d16:0; 18:0)	540.5350	3.26	2.4188	12.959	0.0070	Sphingolipids metabolism (signaling molecule)	Cer
Lactate	89.0244	0.62	2.3795	0.20993	0.0080	Glycolysis	Organic acids
TG(48:0; 5:0)	894.8484	3.76	2.2984	9.0125	0.0107	Lipids metabolism	TG
LPC(18:0)-CH3	508.3409	2.56	2.2979	0.3309	0.0107	Glycerolipids metabolism	GPC
LPE(14:0)	426.2615	1.36	2.2618	0.33441	0.0121	Glycerophospholipids metabolism (energy source)	GPE
Acetylcholine	146.1176	1.41	2.2461	0.23021	0.0127	Glycerophospholipids metabolism	Neurotransmitters
FA(26:0)	395.3894	3.42	2.2333	0.53122	0.0133	Fatty acids metabolism	Fatty acids and conjugates
TG (14:0; 15:0; 16:0)	782.7232	3.62	2.2324	11.318	0.0133	Lipids metabolism (energy source)	TG
L-carnitine	162.1124	0.65	2.194	0.39544	0.0151	Thermogenesis	Carnitine
Oleic acid	281.2487	3.11	2.1839	0.2255	0.0156	Fatty acids biosynthesis (modulator of androgen hormonal profiles and antioxidant compound)	Fatty acids and conjugates
Butanoic acid	87.0452	1.30	2.1475	0.33113	0.0175	Fatty acids biosynthesis	Fatty acids and conjugates
PC(33:2)	744.5548	3.03	2.117	6.9897	0.0193	Glycerophospholipids metabolism	GPC
DG(16:1; 16:1)	582.5092	3.08	2.0808	0.51222	0.0216	Glycerolipids metabolism	DG
TG(14:0; 16:0; 18:0)	824.7702	3.68	2.0674	0.22051	0.0225	Lipids metabolism (energy source)	TG
FA(18:3)	277.2173	2.27	2.0662	4.0863	0.0226	Fatty acids biosynthesis	Fatty acids and conjugates
SM(d40:2)	785.6531	3.09	2.0638	8.4424	0.0228	Sphingolipids metabolism(Signaling molecule)	SM
TG(31:1; 21:2)	874.7858	3.66	2.0541	0.24076	0.0235	Lipids metabolism	TG
SM(d35:2)	759.5658	3.15	2.0536	0.40846	0.0235	Sphingolipids metabolism	SM
Acyl-C5:1	244.1543	1.77	2.0269	0.24579	0.0255	Fatty acids metabolism (energy source)	Fatty acyl carnitine
g-Oxalo-crotonate	159.0307	0.72	1.9839	0.37428	0.0289	Tryptophan metabolism	Fatty acids and conjugates
PC(36:2)	786.6007	3.01	1.9513	0.40854	0.0318	Glycerophospholipids	GPC
						Biosynthesis	
TG(18:1; 18:1; 18:1)	902.8171	3.70	1.9413	0.56415	0.0328	Lipids metabolism	TG
Pentanoate	101.0609	1.78	1.9217	0.2247	0.0347	Fatty acids metabolism (energy storage)	Fatty acids and conjugates
PE(O-16:1; 20:3)	724.5287	3.41	1.9194	2.5093	0.0349	Glycerophospholipids metabolism	GPE
PE(17:2; 21:1)	770.5694	3.06	1.9166	0.35153	0.0352	Glycerophospholipids metabolism	GPE
PI(32:0)	828.5596	2.67	1.9114	0.39002	0.0359	Phospholipids metabolism	GPI
Hex3Cer(d18:1; 14:0)	996.6465	2.61	1.9087	0.39312	0.0360	Sphingolipids metabolism	Cer
TG(31:0; 20:2)	862.7858	3.68	1.8932	7,540.5	0.0376	Lipids metabolism	TG
PC(32:0)	734.5694	2.96	1.8927	5.4942	0.0376	Glycerophospholipids metabolism	GPC
DG(16:0; 16:1)	584.5249	3.15	1.8921	0.52181	0.0377	Glycerolipids and phospholipids metabolism	DG
FA(21:0)	325.3112	3.06	1.8149	0.41988	0.0466	Fatty acids metabolism (membrane stabilizer)	Fatty acids and conjugates
PA(O-22:3; 18:1)	756.5901	3.17	1.7996	2.6825	0.0486	Phospholipids metabolism	PA

^a^ Fold change value refers to the “infertile versus fertile group” change values.

*m/z*, mass-to-charge ratio; Rt, retention time; VIP, variance importance for projection; GPE, glycerophosphoethanolamines; GPC, glycerophosphatidylcholine; GPS, glycerophosphatidylserines; PA, phosphatidic acids; GPI, glycephosphatidylinositols; Cer, ceramides; TG, triglycerides; SM, sphingomyelins; DG, diacylglycerols; Hex3Cer, trihexosylceramides.

**FIGURE 4:**
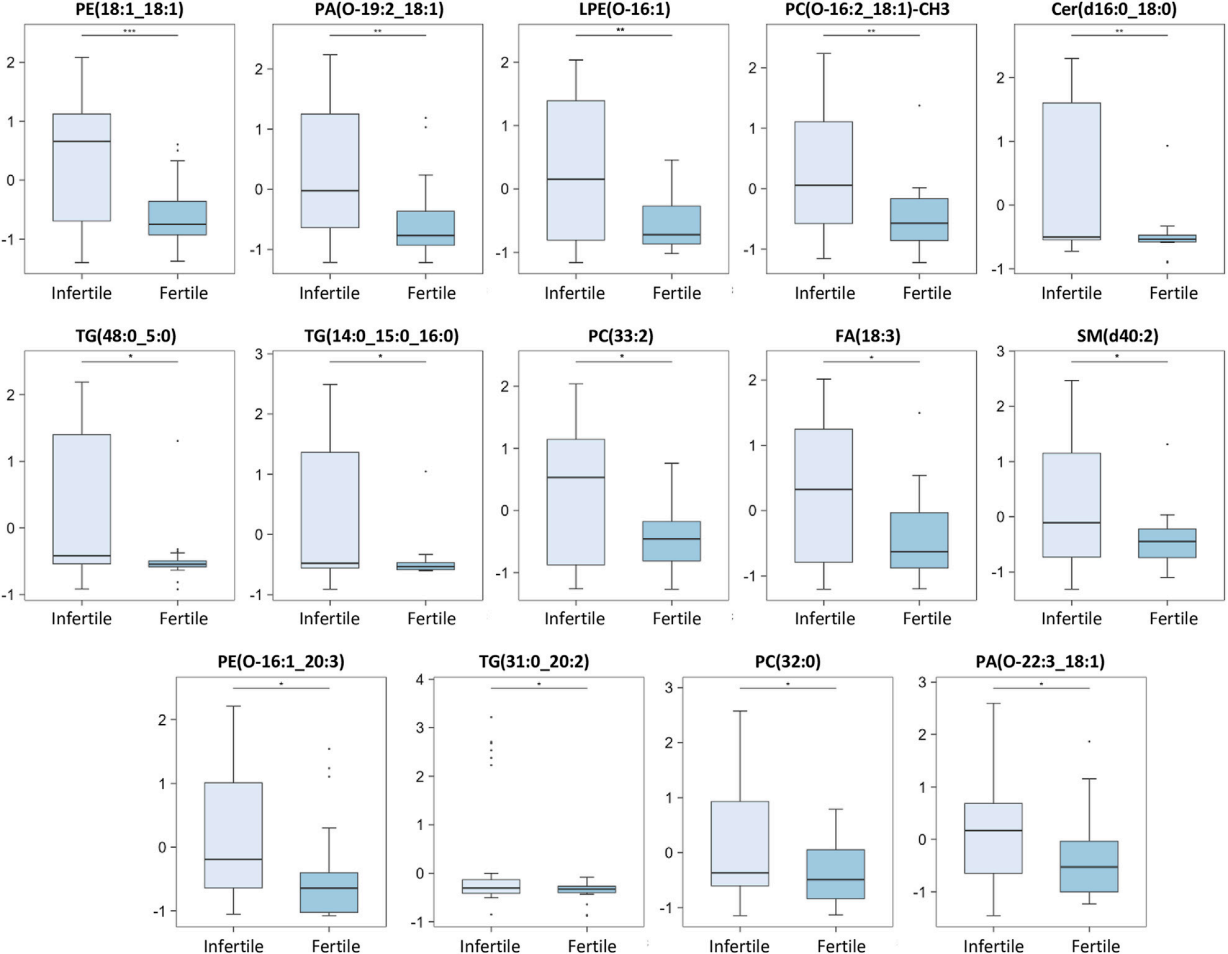
Main discriminant peaks box plot analysis. The box plots show the comparison of the peak intensities of the 14 statistically significant upregulated metabolites and lipids between infertile patients and fertile men. The *p-*values were calculated with Student t-test and the asterisks show the level of significance between the two groups; * *p*-values <0.05, ** *p*-values <0.01, *** *p*-values <0.001. Box plot analysis was performed by RStudio.

**FIGURE 5:**
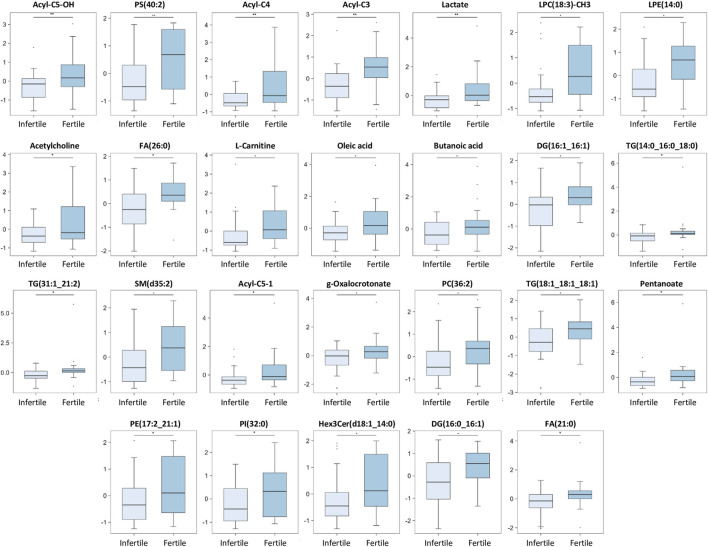
Main discriminant peaks box plot analysis. The box plots show the comparison of the peak intensities of the 26 statistically significant downregulated metabolites and lipids between infertile patients and fertile men. The *p-*values were calculated with Student t-test and the asterisks show the level of significance between the two groups; * *p*-values <0.05, ** *p*-values <0.01. Box plot analysis was performed by RStudio.

A box plot of the normalized peak intensities (autoscale normalized, i.e., mean-centered and divided by the standard deviation of each variable) for each significant compound was also reported in Figures 4, 5 to better evaluate the changes between the two groups.

Our results show that 14 compounds were upregulated (Figure 4) and 26 were downregulated (Figure 5) in infertile patients compared to fertile men. Based on the knowledge of the differential metabolites and upon pathway analysis enrichment against the KEGG pathway database, it can be concluded that the altered metabolic pathways between the fertile and infertile men primarily include glycolysis, glycerophospholipid biosynthesis, fatty acid metabolism, sphingolipid metabolism, thermogenesis, tryptophan metabolism, glycerolipids and phospholipids metabolism (Table 2).

### 3.3 Correlation between potential biomarkers and sperm parameters

**FIGURE 6:**
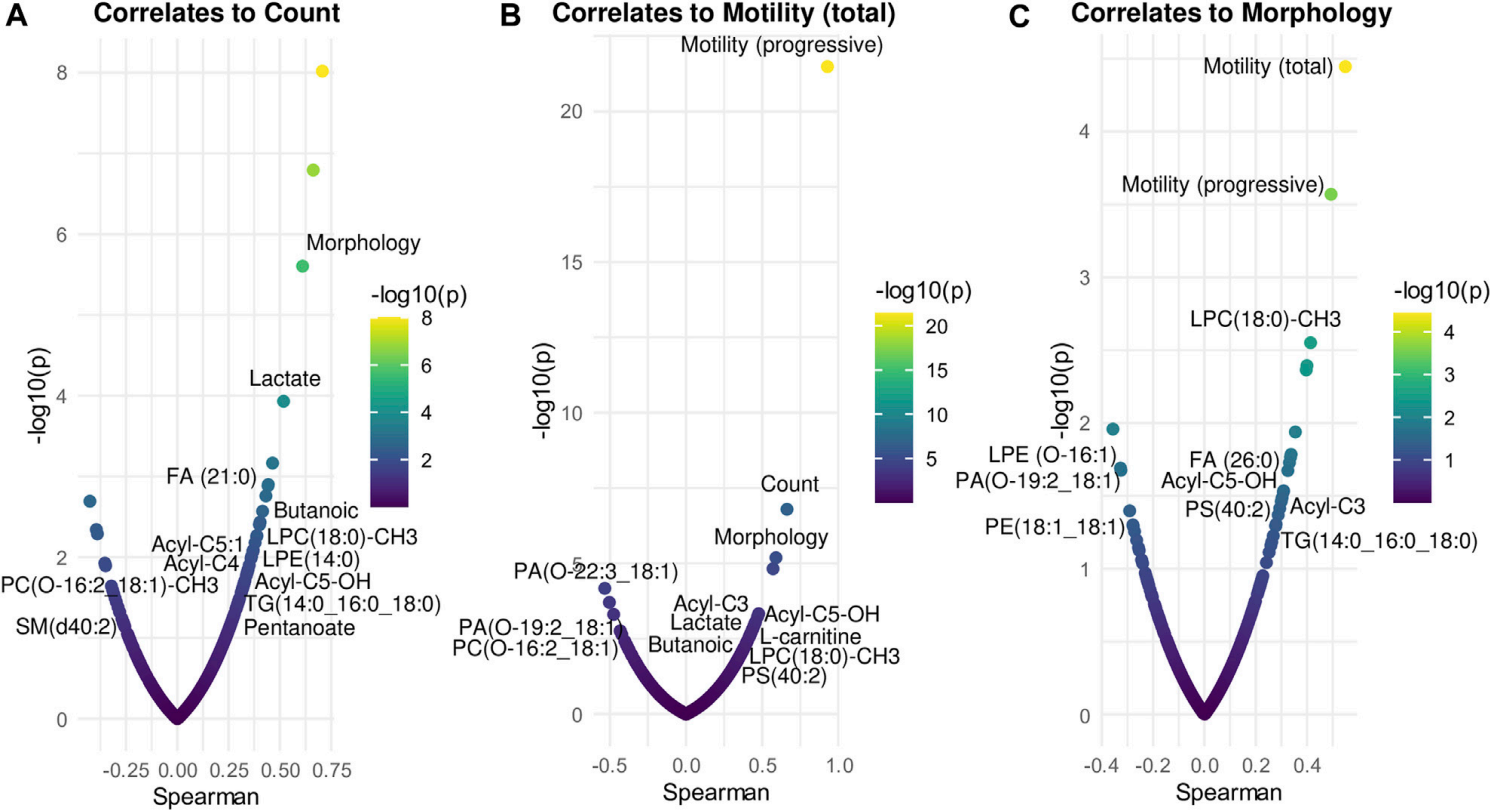
Correlation analysis between compounds and spermiogram parameters. Each panel displays the most significant metabolites and lipids correlated (Spearman’s, *x*-axis) to count **(A)**, total motility **(B)**, morphology **(C)**. The *x*-axis indicates Spearman’s correlation coefficients and the *y*-axis indicates the significance of the correlation (–log_10_ of *p*-values for each correlate). Correlation analysis was performed by RStudio.

To determine the potential clinical relevance of these metabolic and lipidomic findings, the metabolites and lipids levels were correlated to spermiogram parameters (count, total motility and morphology) by Spearman correlation analysis (Figure 6; Supplementary Table S1). In detail, count correlated positively especially with Lactate, FA(21:0), Butanoic acid, LPE(14:0), acyl-C5:1, acyl-C4, acyl-C5-OH, TG(14:0_16:0_18:0), Pentanoate and LPC(18:0)-CH3 while count correlated negatively with PC(O-16:2_18:1)-CH3, SM(d40:2) and PE(O-16:1_20:3) (Figure 6A; Supplementary Table S1). Some of the compounds which correlated positively with sperm count also showed a positive correlation with sperm motility, including Lactate, Pentanoate, LPC(18:0)-CH3, FA(21:0), Butanoic acid, acyl-C5:1, acyl-C4, acyl-C5-OH, LPE(14:0) and TG(14:0_16:0_18:0) (Figure 6B; Supplementary Table S1). In addition, sperm motility was positively associated also with acyl-C3, L-Carnitine, Oleic acid, PS(40:2), FA(26:0), SM(d35:2), TG(18:1_18:1_18:1), PI(32:0), DG(16:0_16:1) while sperm motility was negatively correlated to PE(O-16:1_20:3), PA(O-22:3_18:1), PA(O-19:2_18:1), PC(O-16:2_18:1)-CH3, SM(d40:2), PC(33:2) and Cer(d16:0_18:0) (Figure 6B; Supplementary Table S1). Finally, sperm morphology was found to be positively correlated particularly with LPC(18:0)-CH3, FA(26:0), acyl-C5-OH, PS(40:2), acyl-C3, TG(14:0_16:0_18:0), TG(31:1_21:2) and PE(17:2_21:1) and sperm morphology was found to be negatively associated with LPE(O-16:1), PA(O-19:2_18:1) and PE(18:1_18:1) (Figure 6C; Supplementary Table S1).

### 3.4 Receiver operating characteristic (ROC) analysis

Finally, the selected significant compounds were further analyzed for biomarker analysis to calculate the ROC curve, in order to investigate their specificity and sensitivity. ROC curve is extensively used to evaluate biomarker diagnostic performance, including sensitivity (percentage of true positives identified by the test) and specificity (percentage of true negatives identified by the test), based on the evaluation of the area under the curve (AUC) (Hajian-Tilaki, 2013). The closer the AUC value approaches to 1, the better diagnostic performance the biomarker provides (Hajian-Tilaki, 2013).

**FIGURE 7:**
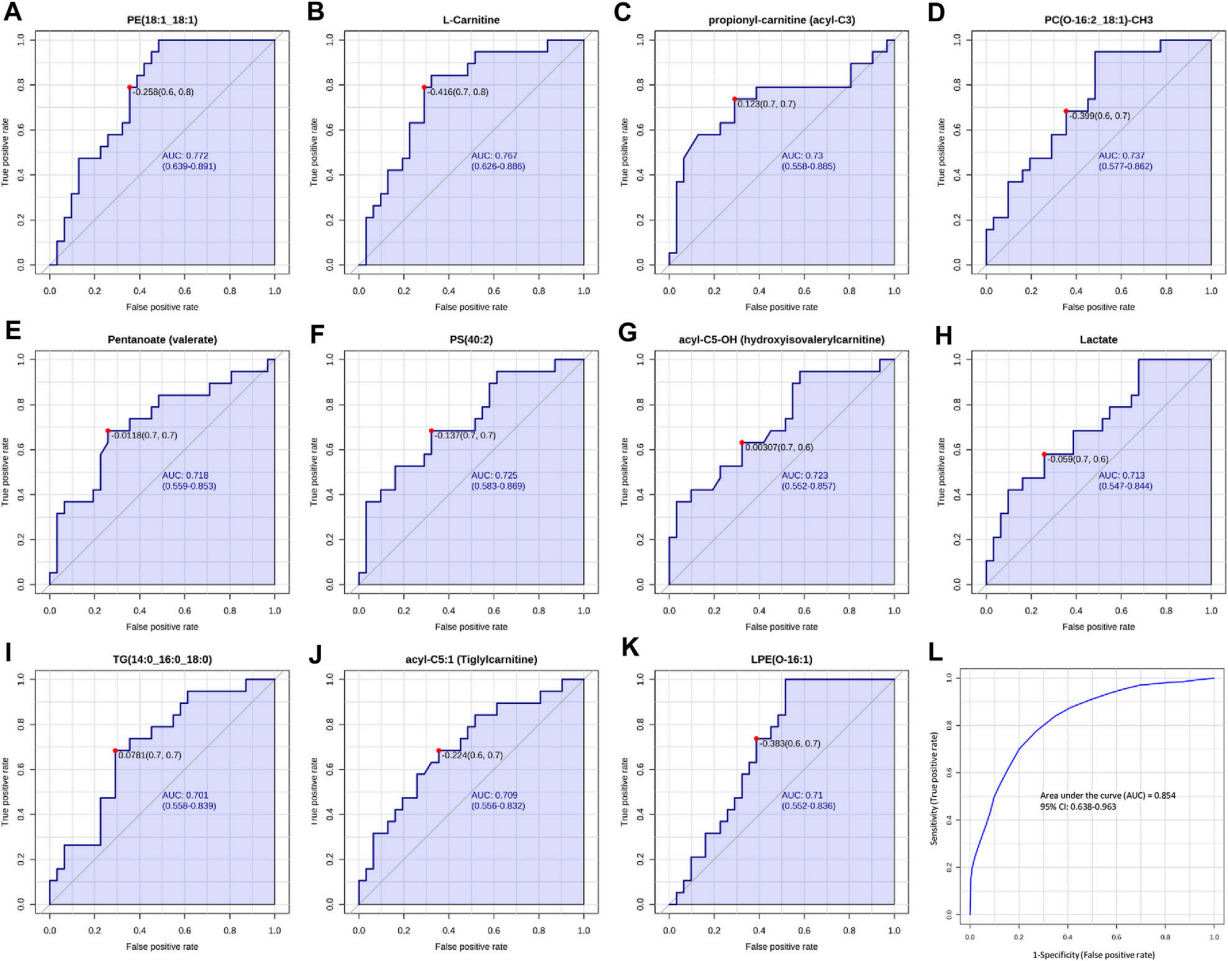
ROC curves analysis of metabolites and lipid compounds between fertile men and infertile patients. **(A–K)** ROC curves of compounds which showed the best diagnostic performances and the ability to discriminate between fertile and infertile men, based on Area under the curve (AUC). **(L)** Cumulative ROC curve analysis, based on the combination of top discriminant compounds. ROC and cumulative ROC curve analysis were acquired by Metaboanalyst software.

Our results showed that, among all the potential biomarkers, PE(18:1; 18:1), L-Carnitine, acyl-C3, PC(O-16:2; 18:1)-CH3, Pentanoate, PS(40:2), acyl-C5-OH, Lactate, TG(14:0; 16:0; 18:0), acyl-C5:1 and LPE(O-16:1) had AUC values around 0.7 (Figures 7A–K), indicating moderate discriminating abilities between fertile and infertile group.

Then a cumulative ROC curve, based on the combination of the above-mentioned compounds, was able to achieve an AUC = 0.848, showing a high sensitivity (0.954) and a moderate specificity (0.629) (Figure 7L). The combination of these potential biomarkers turned out to be a good indicator of the discrimination between fertile and infertile men.

## 4 Discussion

In this study, the integration of untargeted metabolomics and lipidomics analysis of human SP samples was performed aimed at discriminating between fertile and infertile patients by using UHPLC-MS/MS. Contrarily to a targeted approach, which is limited to a specific list of metabolites and lipids, this untargeted approach is addressed to discovery, in order to amplify the repertoire of metabolites/lipids providing new information in this regard. The untargeted approach, while requiring careful scrutiny and validation of each experimental step, clearly provides the best chance to assess novel metabolic perturbations and to identify uncharacterized biomarkers or, even better, a combined biomarker pattern (Gertsman and Barshop, 2018). UHPLC methods coupled to MS significantly improves chromatographic efficiency enabling higher detection sensitivity and resolution separations. As a result, these approaches allow more extensive metabolome coverage than conventional HPLC (Gika et al., 2019).

To the best of our knowledge, this is the first study which analyzed the SP metabolomics and lipidomics MS data of infertile men regardless of the cause of infertility as defined by spermiogram analysis. We compared a heterogeneous group of infertile patients (with different quantitative and/or qualitative alterations of seminal parameters) rather than separated subclasses of infertility conditions. This approach, by discarding the traditional *a priori* infertility classification, could uncover so far undetected subtle metabolite changes able to shed light into the underlying molecular mechanisms of male infertility shared amongst different infertile subgroups.

As shown in Table 2, this study revealed significant changes in specific metabolic pathways associated with infertility conditions. The PLS-DA plots, together with the heatmap as a result of clustering analysis, suggested significant biochemical differences between controls (fertile) and infertile individuals (Figures 2, 3). In particular, a total of 11 metabolites and 29 lipid compounds were found to discriminate infertile from fertile men.

### 4.1 Energy metabolism related metabolites

Different metabolites belonging to the carnitine pathway, were found differentially expressed between fertile and infertile groups. Among these, L-carnitine, acyl-C4, acyl-C3, acyl-C5:1, acyl-C5-OH, were significantly decreased in infertile patients compared to fertile men (Figure 5) and showed a VIP score of 2.194, 2.503, 2.426, 2.027, 2.568, respectively. These metabolites strongly contributed to the effective differentiation between the two groups (Figure 2C; Table 2). These results are in agreement with different MS studies, which compared fertile men and patients with one infertility subtype. For example, Li et al. (2020), Boguenet and others (Boguenet et al., 2020) and Xu et al. (2020) showed significantly reduced concentrations of carnitines in asthenozoospermic, oligoasthenozoospermic and oligoasthenoteratozoospermic patients, respectively compared to healthy controls.

Different studies have already shown the correlation between carnitines and sperm parameters (Gürbüz et al., 2003; Xu et al., 2020; Olesti et al., 2023). In particular, carnitines were positively correlated to sperm number/concentration (Xu et al., 2020; Olesti et al., 2023), sperm motility and morphology (Matalliotakis et al., 2000; Gürbüz et al., 2003). In line with literature findings, our investigation has also demonstrated a positive correlation of carnitines with sperm parameters, including count (acyl-C5:1, acyl-C4, acyl-C5-OH), motility (acyl-C3, acyl-C5-OH, L-carnitine, acyl-C5:1, acyl-C4) and morphology (acyl-C3, acyl-C5-OH)) (Figure 6). During spermatogenesis, the carnitine system plays a vital role in cellular energy metabolism. In fact, it serves not only as a reservoir pool of acyl-coenzyme A but also mediates as a carrier for long-chain fatty acids involved in β-oxidation within fatty acid metabolism (Agarwal and Said,2004). Additionally, carnitines have a prominent role in sperm metabolism and maturation, scavenging free oxygen radicals, reducing apoptosis in spermatogenic cells and inhibiting sperm aggregation (Niederberger, 2005). These findings suggest a significant correlation between carnitines and fertility and can explain the downregulation of carnitines in infertile men observed in our study. In addition, L-Carnitine and L-acetylcarnitines have gained significant recognition as valuable supplements in clinical practice. Patients treated with carnitine alone or in association with other medications, showed an improvement of sperm motility and morphology even in male idiopathic infertility (Khaw et al., 2020). Other *in vitro* studies have demonstrated that the addition of L-carnitine to spermatozoa enhances their vitality and motility (Banihani et al., 2012). Building upon these findings, it has been hypothesized that incorporating L-carnitine into semen samples intended for cryopreservation and assisted reproductive techniques could be beneficial in improving semen quality (Banihani et al., 2014). The utilization of carnitines as valuable supplements in clinical practice could be useful to develop and provide in the future a personalized and effective treatment of infertile men.

Another metabolite able to differentiate infertile from fertile men in our study was lactate. Lactate is produced at the end of the glycolytic pathway through the action of the lactate dehydrogenase (LDH) enzyme (Jelks et al., 2001). In mature spermatozoa, glycolysis is essential for ATP generation (Miki et al., 2004). Moreover, developing germ cells, utilize lactate as unique energy because of their inability to metabolize glucose (Dias et al., 2014). Our data demonstrated that lactate was one of the most important metabolites for group segregation, as a statistically significant decrease was observed in infertile patients compared to fertile controls (Figure 5; Table 2). A positive correlation between lactate and sperm count and motility (Figure 6) was also found in our analysis, supporting the crucial importance of this metabolite for sperm functions. This finding was confirmed by other ^1^H NMR studies in which reduced levels of lactate were found in oligozoospermic (Gupta et al., 2011), obstructive azoospermic (Hamamah et al., 1993) and in OAT patients (Mumcu et al., 2020), compared to normozoospermic men. In contrast, a ^1^H NMR investigation by Mehrparvar et al. (2020), found increased level of lactate in teratozoospermic patients compared to fertile men. Chen and others also detected enhanced level of lactate in asthenozoospermic patients compared to normozoospermic by using UHPLC-MS (Chen et al., 2020).

These contrasting findings in the levels of lactate between fertile and infertile men, could originate from the use of differential techniques for metabolomic and lipidomic analysis (H-NMR or MS), distinct processing and sample preparation methods and also heterogeneity or small-sample size of fertile vs. infertile groups. Obviously, all these variables can lead to differences in study results. However, altogether these studies consistently point at dysregulated carnitine metabolism and lactate generation/consumption rates. As lactate uptake and consumption is impaired in dysfunctional mitochondria, and acyl-carnitine metabolism is a hallmark of mitochondrial dysfunction in the context of altered fatty acid oxidation, these results are overall consistent in pointing out a role for sperm mitochondrial dysfunction in male infertility. Of note, elevation of both classes of metabolites in bodily fluids are a hallmark of mitochondrial dysfunction in patients with long-COVID (Guntur et al., 2022; López-Hernández et al., 2023), contributing a reasonable rationale underpinning the rise in male infertility following the SARS-CoV-2 global pandemic (Hu et al., 2022).

### 4.2 FAs related metabolites

FAs play a key role in sperm development and maturation, in the regulation of fertilization process, sperm membrane fluidity and exhibit antioxidant functions (Shan et al., 2021). Some studies have explored the connection between fertility and dietary fatty acids (Wahes et al., 2007; Esmaeili et al., 2015), shedding light on their potential therapeutic purposes as nutritional supplements.

Our data revealed Butanoic/Butyric acid and pentanoate as potential discriminative biomarkers between fertile and infertile men also showing a positive correlation with sperm parameters (Figures 5, 6). In particular, decreased levels of Butanoic/Butyric acid and Pentanoate, with a VIP score of 2.148 and 1.922 respectively (Figure 2C; Table 2), were found in infertile patients compared to fertile men (Figure 5); these metabolites were also positively associated with sperm count and motility (Figure 6; Supplementary Table S1). In agreement with our data, Tang et al. (2017) showed a reduction of butanoic acid in the SP of asthenozoospermic patients compared to healthy controls.

To the best of our knowledge, no previous studies reported a statistically significant difference of pentanoate in the SP from fertile and infertile men, nor its significant correlation with sperm parameters, which is worth for further investigations to better understand its implication in male infertility.

Butanoic/Butyr acid and Pentanoate are short-chain fatty acids (SCFAs), that are produced through the fermentation of dietary fiber by gut bacteria in the colon (Koh et al., 2016; Luu et al., 2019). In particular, Butyrate has been shown to inhibit histone deacetylase (HDAC) and to influence sperm movement in rats (Parab et al., 2015). Additionally, in mice, butyric acid plays a crucial role in the modulation of transcription process during the transition from meiotic to post-meiotic in spermatogenesis (Yin et al., 2021). Furthermore, butyrate is involved in maintaining the redox balance in rats (Lin et al., 2014), which is vital for spermatogenesis (Sênos Demarco and Jones, 2021). A recent study conducted on boars revealed that dietary fiber supplementation enhances gut microbiota and increases the production of total short-chain fatty acids, including butyric acid (Lin et al., 2022). This, in turn, improves spermatogenesis and semen quality (Lin et al., 2022). These preliminary studies on animal models might suggest the potential role of Butyric acid in fertility impairment and its nutritional implications as a supplement to enhance sperm parameters in infertility.

A statistically significant decreased level of oleic acid was found in infertile group compared to fertile one (Figure 5; Table 2) and a positive correlation with sperm motility was also observed (Supplementary Table S1). In support of our results, Wang and others reported that oleic acid was significantly correlated with a lower risk of low semen quality (Wang et al., 2019). In addition, Xu and others detected reduced levels of oleic acid in oligoasthenoteratozoospermic patients when compared to healthy controls (Xu et al., 2020). Spermatozoa membrane contains very high levels of several unsaturated FAs (such as oleic acid, arachidonic acid), which are crucial for sperm motility and membrane fluidity (Safarinejad et al., 2010). In contrast, Tang et al. (2017) reported an increased level of oleic acid in asthenozoospermic men compared to healthy controls, by using GC/MS. However, these contradictory results in the levels of oleic acid between fertile and infertile men, could be due to samples selection: Tang and others focused on asthenozoospermic men while this study analysed infertile subjects regardless of the semen subtype classification.

Oleic acid, the main constituent of olive oil, seems to have a role in preserving semen quality in humans (Saleem et al., 2021). In fact, administration of extra virgin oil in male volunteers with poor semen quality was found to have a positive effect on the androgen hormonal profiles, by increasing the testosterone and luteinizing hormone levels (Derouiche et al., 2013). It has also been reported that oleic acid has antioxidant properties (Reaven et al., 1993), enabling the neutralization of free radicals which could damage sperm membrane and preventing lipid peroxidation properties (Reaven et al., 1993). These defense properties of oleic acid may improve sperm quality, suggesting its utility as diet supplementation for the therapeutic treatment of male infertility.

A number of compounds in our study were found to be decreased in a statistically significant level in infertile group compared to fertile men. These include Oxalo-crotonate, FA (26:0) and FA(21:0) (Figure 5; Table 2). To the best of our knowledge, this is the first report, which demonstrated significantly reduced levels of these lipid metabolites in the SP samples of infertile men. Interestingly, the discovery of so far undetected dysregulation of these lipids in male infertility arises, maybe, from the novelty of our approach to classify in the same group infertile men with different kind of semen disorders. Obviously, the significance of these findings warrants further investigations.

On the other hand, FA(18:3) or α-linolenic acid, an omega-3 polyunsaturated fatty acid (PUFA), was found to be increased in a statistically significant level in infertile group compared to fertile men (Figure 4). Numerous studies in the literature have highlighted the crucial significance of PUFAs in sperm biology, in spermatogenesis, and in reproductive processes (Collodel et al., 2020). In agreement with our results, Gulaya and others showed that FA(18:3) was increased in semen of infertile men, and was negatively correlated with sperm motility (Gulaya et al., 2001).

Of note, it has been reported that an excess of PUFA, represents a biochemical signature of defective spermatozoa together with high reactive oxygen species (ROS) generation (Ollero et al., 2001). In fact, when spermatozoa reside in the testis, they are characterized by high PUFA levels, which are normally lost during the functional remodeling of the sperm membrane occurring in the epididymal transit (Aitken et al., 2006). Therefore, the persistence of elevated PUFA levels could probably be due to a failure of sperm maturation, often connected with the excess of residual cytoplasm and the excessive generation of ROS. In fact, it should be noted that elevated PUFA levels cause spermatozoa sensitivity to ROS attack, which could promote a cascade of chemical reactions known as lipid peroxidation (Shan et al., 2021). In light of the above considerations, PUFA species require much more attention as putative markers of sperm oxidation.

### 4.3 Phospholipids related metabolites

Different PC species [PC(O-16:2; 18:1)-CH3, PC(32:0), PC(33:2)] were found to be significantly higher in infertile patients when compared to fertile men (Figure 4).

Choline and its derivatives not only serve as vital components of cell membranes but also play an important role in the transport and metabolism of lipid cholesterol (Zeisel and da Costa, 2009). In addition, abnormalities in the phospholipids metabolism may affect different biological processes, for example, inflammation (Feingold and Grunfeild, 2022). The increased levels of choline and PC in the SP of infertile patients therefore could suggest an altered choline metabolism associated with infertility. Interestingly, PC(O-16:2; 18:1)-CH3 showed a negative association with both sperm motility and count, while PC(33:2) showed a negative association with sperm motility only (Figure 6; Supplementary Table S1). In agreement with this finding, Engel and others (Engel et al., 2019) revealed a negative correlation between PC and sperm concentration. In contrast, (Boguenet et al., 2020) found a decrease in some PC species in the SP of severe oligoasthenospermic patients compared with men with normal semen parameters. These discrepancies could arise from differences in the cohort selection: Boguenet and others focused on oligoasthenozoospermic patients while this study analysed infertile subjects regardless of the semen subtype classification.

Significantly increased levels of PE and LPE compounds (PE (18:1; 18:1), PE (O-16:1; 20:3)), LPE(O-16:1), were found in infertile compared to fertile group (Figure 4). Among these, PE(18:1; 18:1) and LPE(O-16:1) were negatively correlated with sperm morphology, suggesting a possible involvement of these specific lipids in certain sperm abnormalities (Figure 6).

While the physiological functions of LPC in reproduction have been reported, including the deterioration of spermatozoal plasma membrane associated with an increase of sperm LPC (Glander et al., 2002), to the best of our knowledge, this is the first report documenting such a dramatic increase of PE, in particular PE(18:1; 18:1), in the SP of infertile subjects. These findings warrant further investigations.

In this investigation, PS(40:2) was found significantly decreased in infertile patients (Figure 5). PS are known to be involved in apoptotic cell events through the process of PS membrane translocation, which is recognized as an important biological event in the cell (Kotwicka et al., 2013). It was suggested that PS membrane translocation could be responsible for the changes in the spermatozoa cell membrane which happen during capacitation and acrosome reaction or during the elimination of pathological spermatozoa (Kotwicka et al., 2013). In fact, a significant decrease of the vital spermatozoa with PS membrane translocation after swim-up selection was demonstrated (Kotwicka et al., 2013). Of note, a recent study by Rival et al. (2019), demonstrated that PS, exposed on viable sperm, is recognized by specific receptors on oocytes, promoting sperm:egg fusion and playing a key role during fertilization process. In fact, they also observed that fertilization can be compromised when PS is blocked on sperm or when certain PS receptors on the oocytes are blocked or lost (Rival et al., 2019). Therefore, the significantly decreased levels of PS, which we have observed in infertile patients (Figure 5), may not only affect sperm motility and morphology (Figure 6), but also negatively affect the fertilization process (Rival et al., 2019) thus contributing to the infertility condition of the recruited subjects by two different mechanisms.

The present investigation also showed significantly increased levels of some PA molecules (PA(O-19:2; 18:1), PA(O-22:3; 18:1)) in infertile compared to the fertile group (Figure 4). Among these, PA(O-19:2; 18:1) was negatively correlated with both sperm motility and morphology while PA(O-22:3; 18:1) showed a negative correlation with sperm motility only (Figure 6).

It has been reported that PA is involved the production of ROS by activating NADPH oxidase (Palicz et al., 2001). As it is known that excessive release of ROS plays an essential role in the inflammation process, it is tempting to speculate that the significantly increased levels of PA we have observed may play a causative role in the condition of infertility of the subjects recruited in this study. Since PA also participates in the coordination of mitochondrial dynamics (Kameoka et al., 2018), this result may contribute to the depiction of a dysfunctional mitochondrial phenotype in infertile sperm.

Increased levels of a Sphingomyelin (SM) molecule SM(d40:2) and Cer(d-16:0; 18:1), were also found in infertile group compared to fertile men (Figure 4). These results are in line with the study of Xu et al. (2020), which showed significantly higher level of an SM molecule in the SP of the infertile group, despite identifying a different SM molecule in their investigation. Interestingly, a negative correlation between SM(d40:2) and sperm count and motility was also reported in our study (Figure 6; Supplementary Table S1). Boguenet et al. (2020) found a decrease in some SM species in the SP of severe oligoasthenospermic patients compared with men with normal semen parameters. Again, as mentioned above, these contrary findings might derive from differences in patients selection: Boguenet and others focused on oligoasthenozoospermic patients while this study analysed infertile subjects regardless of the semen subtype classification. Sphingolipids play crucial roles in various cellular processes such as cell differentiation, apoptosis and steroid production (Wang et al., 2023).

Some investigations have also unveiled the involvement of sphingolipid metabolites in the generation of spermatozoa and the regulation of germ cells apoptosis caused by stress or damage (Tilly and Kolesnick, 1999; Morita and Tilly, 2000). These findings suggest a potential connection between sphingolipids and the impairment of gonadal function and infertility. Interestingly, Cer is involved in the process of germ cell death (Suomalainen et al., 2003). Therefore the significantly increased levels of Cer(d-16:0; 18:1) in infertile individuals might again play a contributing role in the condition of infertility of the subjects recruited in this study.

### 4.4 Glicerolipids related metabolites

In this investigation, different TG species [TG(14:0; 16:0; 18:0), TG(31:1; 21:2), TG(18:1; 18:1; 18:1)] and some diglycerides (DG) molecules [DG(16:1; 16:1), DG(16:0; 16:1)] were found significantly decreased in infertile patients (Figure 5).

In a previous study, decreased levels of TG and DG molecules were detected in spermatozoa of asthenozoospermic patients compared to healthy men (Chen et al., 2021). Interestingly, TG(14:0; 16:0; 18:0) also showed a positive correlation with all the seminal parameters, sperm count, motility and morphology (Figure 6; Supplementary Table S1), suggesting its potential correlation with specific spermatozoa defects.

Diglycerides (or diacylglycerols) have been reported to be involved in the acrosome reaction and in membrane fusion during acrosomal exocytosis in ram and humans (Roldan and Murase, 1994; O’Toole et al., 1996). Interestingly, a previous study highlighted a significant association between reduced DG levels in sperm and bull idiopathic infertility (Shan et al., 2020). These findings support our data and underscore the potential importance of DAGs in male reproductive health.

### 4.5 Other metabolites

As far as we are aware, our study showed, for the first time, significantly decreased levels of Acetylcholine in the human SP from infertile patients when compared to fertile individuals (Figure 5; Table 2). It has been reported that Acetylcholine, at physiological levels, plays an important role in spermatogenesis and sperm motility in human and mice (Bray et al., 2005). This neurotransmitter is also important for penile erection and relaxation in humans and it is also correlated with the central control of ejaculation (Sadia et al., 2023). A very recent study showed an increase in blood acetylcholinesterase activity in infertile males, corroborating the involvement of cholinergic system in infertility conditions. Acetylcholinesterase has also been observed in seminal fluid, human prostrate, and sperm and its presence is linked with sperm motility (Nieto-Cerón et al., 2010; Ammar et al., 2019). Ammar and others revealed increased activity of this enzyme in the SP of teratozoospermic patients founding also an association with apoptosis (Ammar et al., 2019). It is reported that acetylcholinesterase is even responsible for the reduced level of testosterone in human and rat Leydig cells (Favaretto et al., 1993; Gupta, 2011). Together these findings, induces us to hypothesize that the reduced levels of acetylcholine observed in our infertile patients, could be explained by an increased activity of acetylcholinesterase in sperm, which could lead to the lower availability of the neurotransmitter for physiological roles because of its rapid hydrolysis.

In summary, all these findings highlight the significance of maintaining appropriate levels of Acetylcholine for healthy sperm development and function, while emphasizing the potential negative effects of abnormal concentrations of this neurotransmitter on male reproductive health.

### 4.6 Multiple biomarkers selection by ROC analysis and diagnostic perspectives

Our findings enhance and expand the currently available information in the metabolomics and lipidomics field of the SP, by providing novel insights and a more detailed and refined understanding of the altered metabolic pathway involved in male infertility. In fact, different compounds, including L-carnitine, propionyl-l-carnitine, acyl-C5:OH and acyl-C5:1, Lactate, Pentanoate, PE(18:1; 18:1), PC(O-16:2; 18:1)-CH3, LPE(O-16:1), PS(40:2) and TG(14:0; 16:0; 18:0) showed a good sensitivity by ROC analysis in the discrimination between fertile and infertile men (Figure 7). Of note, a combination of biomarkers could provide higher predictive power than single ones. The resulting ROC curve from the combinations of the above mentioned top nine biomarkers (L-carnitine, propionyl-l-carnitine, acyl-C5:OH and acyl-C5:1, Lactate, Pentanoate, PE(18:1; 18:1), PC(O-16:2; 18:1)-CH3, LPE(O-16:1), PS(40:2) and TG(14:0; 16:0; 18:0) (Figure 7L) showed the highest AUC = 0.854, with sensitivity and specificity equal to 96.3% and 63.8%, respectively. The ROC model revealed a satisfactory improved diagnostic ability, demonstrating that the combined biomarker pattern could better characterize the global perturbation of the metabolomics network in infertility patients. Although providing an interesting metabolic insight, these results are preliminary and need to be further validated on larger study cohorts.

To the best of our knowledge, this is the first LC-MS-based report which found significant variations of a subset of metabolites and lipid compounds in the SP samples from fertile and infertile patients, including acetylcholine, pentanoate, g-oxalocrotonate, TG, DG, Cer, PA, PI, PS.

Interestingly, some of the mentioned statistically significant molecules showed common correlations with sperm parameters. Among these, acyl-C5-OH, TG (14:0; 16:0; 18:0) and LPC(18:0)-CH3 besides having a good sensitive by ROC analysis, were also positively associated with all seminal parameters, sperm count, motility and morphology (Figure 6; Supplementary Table S1), suggesting that these molecules could serve as promising candidate biomarkers for male infertility. For these three molecules in detail, and in general in all the cases in which a lower metabolite concentration has been found in infertile subjects in comparison to fertile controls, metabolites can be supplemented directly from diet. Therefore, a specific nutrition planning based on evaluation of patient’s diet might be an appropriate targeted therapeutic intervention for the treatment and the management of male infertility.

Our study holds several limitations, first of all the relatively small sample size used for the multivariate statistical analysis; indeed, a larger cohort of patients will be required to prospectively validate the underlying pathways and networks as well as the validity of potential discriminating candidates for male infertility conditions. Despite the limitations of this study, our data improve currently available metabolomics and lipidomics information on SP and highlights the importance of data integration for a better understanding of pathophysiological mechanisms responsible for fertility impairment.

As a next step, fertile subjects with abnormal semen analysis or infertile subjects with normal semen analysis will be enrolled in order to investigate the potential involvement of these biomarkers in idiopathic infertility.

In conclusion, using a UHPLC-MS based untargeted approach, metabolomics/lipidomics profiling of SP from fertile and infertile men was performed, regardless sub-infertility status. Unique SP metabolomic and lipidomic features were found in infertile patients compared to fertile. In particular, we found a panel of 11 SP metabolites able to differentiate fertile from infertile and which belonged to different pathways, including cell energy metabolism, glycolysis, glycerophospholipid biosynthesis and fatty acid metabolism and a panel of 29 lipid compounds, including PE, PA, LPE, PC, PS, LPC, FA, TG, SM, DG, Cer (Table 2). Although many of the potential biomarkers (L-carnitine, acylcarnitines, Lactate, Butanoic acid, oleic acid, PC, PE, LPE, PS) of male infertility identified in our study were already reported in previous metabolomics investigations, these biomarkers were related only to a specific subclass of infertility. These findings demonstrated that our pilot metabolomics approach might be strongly predictive for male infertility diagnosis providing a more accurate picture of metabolic and lipid dysregulations associated to infertility disorders. Consequently, this approach might suggest the appropriate therapeutic intervention. Despite these findings suggest potential biomarkers with diagnostic relevance, significant limitations of this study are the small sample size and the lack of external validation. Indeed, recruiting additional subjects has already started in order to confirm these findings on larger cohorts. If validated in larger and diverse cohorts, the biomarkers here identified could contribute to the development of novel strategies to diagnose male infertility and they could also provide the theoretical basis for the development of new therapies for the management of such unmet medical need. Therefore, this study can act as a first milestones towards it.

## Data Availability

The original contributions presented in the study are included in the article/Supplementary Material, further inquiries can be directed to the corresponding authors.
