# Supplementary material for: Seminal plasma untargeted metabolomic and lipidomic profiling for the identification of a novel panel of biomarkers and therapeutic targets related to male infertility
Source: Front Pharmacol. 2023 Sep 27;14:1275832. doi: 10.3389/fphar.2023.1275832 (PMC10565040; doi:10.3389/fphar.2023.1275832)
Supplement: Supplementary file 1 [file Table1.DOCX]

Supplementary Material

**Supplementary Table S1**. Correlation analysis data between compounds and sperm parameters.

| **Compounds** | **Sperm count** | | **Sperm motility** | | **Sperm morphology** | |
| --- | --- | --- | --- | --- | --- | --- |
|  | **Spearman’s correlation coefficients** | **Significance of the correlation** | **Spearman’s correlation coefficients** | **Significance of the correlation** | **Spearman’s correlation coefficients** | **Significance of the correlation** |
| **PE(18:1;18:1)** | Not correlated | Not significant | Not correlated | Not significant | -0.279 | 1.30 |
| **PA(O-19:2;18:1)** | Not correlated | Not significant | -0.431 | 2.76 | -0.325 | 1.67 |
| **LPE(O-16:1)** | Not correlated | Not significant | Not correlated | Not significant | -0.327 | 1.69 |
| **PC(O-16:2;18:1)-CH3** | -0.350 | 1.89 | -0.402 | 2.42 | Not correlated | Not significant |
| **Acyl-C5-OH** | 0.340 | 1.80 | 0.452 | 3.01 | 0.309 | 1.53 |
| **PS(40:2)** | Not correlated | Not significant | 0.340 | 1.80 | 0.308 | 1.53 |
| **Acyl-C4** | 0.350 | 1.90 | 0.332 | 1.73 | Not correlated | Not significant |
| **Acyl-C3** | Not correlated | Not significant | 0.456 | 3.05 | 0.293 | 1.41 |
| **Cer(d16:0;18:0)** | Not correlated | Not significant | -0.360 | 1.99 | Not correlated | Not significant |
| **Lactate** | 0.518 | 3.93 | 0.449 | 2.97 | Not correlated | Not significant |
| **LPC(18:0)-CH3** | 0.400 | 2.40 | 0.382 | 2.21 | 0.414 | 2.55 |
| **LPE(14:0)** | 0.370 | 2.09 | 0.317 | 1.60 | Not correlated | Not significant |
| **FA(26:0)** | Not correlated | Not significant | 0.358 | 1.97 | 0.338 | 1.78 |
| **L-carnitine** | Not correlated | Not significant | 0.438 | 2.83 | Not correlated | Not significant |
| **Oleic acid** | Not correlated | Not significant | 0.375 | 2.14 | Not correlated | Not significant |
| **Butanoic acid** | 0.415 | 2.57 | 0.399 | 2.39 | Not correlated | Not significant |
| **PC(33:2)** | Not correlated | Not significant | -0.333 | 1.74 | Not correlated | Not significant |
| **TG(14:0;16:0;18:0)** | 0.329 | 1.71 | 0.337 | 1.78 | 0.279 | 1.30 |
| **SM(d40:2)** | -0.315 | 1.59 | -0.376 | 2.14 | Not correlated | Not significant |
| **TG(31:1;21:2)** | Not correlated | Not significant | Not correlated | Not significant | 0.299 | 1.46 |
| **SM(d35:2)** | Not correlated | Not significant | 0.368 | 2.07 | Not correlated | Not significant |
| **Acyl-C5:1** | 0.359 | 1.98 | 0.339 | 1.80 | Not correlated | Not significant |
| **TG(18:1;18:1;18:1)** | Not correlated | Not significant | 0.286 | 1.36 | Not correlated | Not significant |
| **Pentanoate** | 0.303 | 1.49 | 0.341 | 1.82 | Not correlated | Not significant |
| **PE(O-16:1;20:3)** | -0.426 | 2.69 | -0.503 | 3.70 | Not correlated | Not significant |
| **PE(17:2;21:1)** | Not correlated | Not significant | Not correlated | Not significant | 0.325 | 1.67 |
| **PI(32:0)** | Not correlated | Not significant | 0.336 | 1.77 | Not correlated | Not significant |
| **DG(16:0;16:1)** | Not correlated | Not significant | 0.304 | 1.50 | Not correlated | Not significant |
| **FA(21:0)** | 0.442 | 2.88 | 0.470 | 3.24 | Not correlated | Not significant |
| **PA(O-22:3;18:1)** | Not correlated | Not significant | -0.533 | 4.17 | Not correlated | Not significant |

PE, Phosphoethanolamine; PA, Phosphatidic acid; LPE, Lysophosphoethanolamine; PC, Phosphatidylcholine; PS, Phosphatidylserine; Cer, ceramides; LPC, Lysophosphatidylcholine; TG, Triglycerides; SM, Sphingomyelins; PI: Phosphatidylinositol; DG, Diacylglycerols.
